# Untargeted Profiling and Differentiation of Volatiles in Varieties of Meat Using GC Orbitrap MS

**DOI:** 10.3390/foods11243997

**Published:** 2022-12-09

**Authors:** Youyou Yang, Jing Li, Jiangtao Xing, Weihai Xing, Chaohua Tang, Zhenghua Rao, Junmin Zhang

**Affiliations:** 1 State Key Laboratory of Animal Nutrition, Institute of Animal Sciences of Chinese Academy of Agricultural Sciences, Beijing 100193, China; 2 Scientific Observing and Experiment Station of Animal Genetic Resources and Nutrition in North China of Ministry of Agriculture and Rural Affairs, Institute of Animal Sciences of Chinese Academy of Agricultural Sciences, Beijing 100193, China; 3 Thermo Fisher Scientific, 8/F, Tower C, Global Trade Center, No. 36, Beisanhuan East Road, Dongcheng District, Beijing 100013, China

**Keywords:** differentiation, GC-HRMS, SPME, statistical analysis, untargeted profiling, volatiles

## Abstract

Volatile compounds play vital roles in food sensory attributes and food quality. An analysis of volatile compounds could illustrate the sensory attributes at the microscale level. Here, untargeted profiling approaches for volatiles in five most-consumed meat species were established using headspace SPME-GC/high resolution Orbitrap MS. An extended high-resolution database of meat volatile compounds was established to enhance the qualification accuracy. Using sulfur-containing compounds, aldehydes, and ketones as the research model, the parameters including fiber coating types, extraction temperature, extraction time, and desorbing time were optimized. Principle component analysis, volcano analysis and partial least squares discriminant analysis were applied to run the classification and the selection of discriminant markers between meat varieties, respectively. Different varieties could be largely distinguished according to the volatiles’ profiles. 1-Octen-3-ol, 1-octen-3-one, 2-pentyl furan and some other furans degraded from n-6 fatty acids would contribute to distinguishing duck meat from other categories, while methyl esters mainly from oleic acid as well as dimethyl sulfoxide and carbon disulfide possibly produced from the sulfur-containing amino acids contributed to the discrimination of beef. Therefore, volatiles’ profiling not only could interpret the aroma style in meat but also could be another promising method for meat differentiation and authentication.

## 1. Introduction

Meat quality includes safety, nutrition, and sensory aspects, which may be largely affected by multiple factors such as heredity, breeding, and the environment [1]. With the improvement in living standards, consumers place more and more emphasis on the nutrition and flavor. Flavor consists of aroma and taste [2]. Aroma, an important part of sensory evaluation, has become one of the important determinants influencing consumers’ purchasing choices.

Chemical composition is the basis of food aroma. With the maturation of analytical techniques and rapid development of instrumentation, more and more aroma compounds and their contributions have been discovered. Constituting the unique style of aroma, aroma compositions are complex, mainly including alcohols, esters, organic acids, ketones, aldehydes, furans, pyrazines, hydrocarbons, sulfur-containing compounds [3,4], and have very wide ranges of polarity, solubility, volatility, and thermal stability. This would bring a big challenge to the untargeted profiling of the aroma compounds. Basically, the analysis of aroma compounds includes three steps: extraction, separation, and identification.

Headspace solid phase microextraction (HS-SPME) [3,4,5,6,7], solvent-assisted flavor evaporation (SAFE) [8], stir bar adsorption extraction (SBSE) [9], and dynamic headspace (DHS) [10] are the mainstream extraction methods. SAFE is considered as the mild solvent-extraction technique without discrimination of volatile compounds. It is usually used to profile and quantitate volatile compounds accurately, showing good potential in aroma reformulation. HS-SPME and SBSE are solvent-free micro-extraction technologies, which integrate sampling, extraction, and concentration [11]. They could directly extract analytes from complex matrices and have been widely used for differential analysis of aroma compounds. The adsorption competition existed in both SPME and SBSE. The extraction efficiencies are largely affected by coating types, extraction time, and extraction temperature [12,13,14,15]. Compared with SPME, the coating in SBSE has a larger surface area and more adsorption sites [16]; thus, the adsorption capacity in SBSE could be enhanced [17,18]. However, the full automation could not be realized in both SAFE and SBSE, which could largely confine the analysis throughput. Considering the throughput, SPME would be a promising extraction method for profiling.

Regarding the separation of aroma compounds, gas chromatography-ion mobility spectrometry and GC-MS are mostly utilized [3,19]. MS could give the structural information with good identification capability, making it the dominant technique in the aroma research area. The introduction of high-resolution mass spectrometry increases the mass accuracy of the fragments and greatly improves the identification accuracy [20]. In order to increase the peak capacity, two-dimensional gas chromatography [21,22] was also introduced and could reduce the co-elution phenomenon, significantly increasing the volume of volatile compounds identified. Until now, the identification of aroma compounds has been mainly based on the MS pattern and retention index (RI) in NIST and Wiley libraries. However, RI could be slightly varied with different types of different specifications of columns, respectively. Additionally, different MS detectors could largely influence the MS patterns since orbitrap and quadrupole have different detection principles. This would affect the identification accuracy and cause false positive or false negative results when libraries are mismatched.

Accurate aroma analysis is quite important for meat quality research and the build-up of micro-correlation between the meat sensory attributes and the aroma compounds. In this study, GC combined with high-resolution orbitrap MS and SPME was utilized to profile volatile compounds and differentiate various meat categories such as pork, beef, mutton, chicken, and duck breast with statistical analyses tools.

## 2. Reagents and Materials

### 2.1. Chemicals

Analytical standards of benzene, toluene, styrene, p-cymene, o-xylene, dl-limonene, 1-pentanol, 1-hexanol, 1-octen-3-ol, 1-decanol, α-terpineol, benzenemethanol, benzeneethanol, 3-methylphenol, methyl octanoate, ethyl acetate, methyl butanoate, vinyl hexanoate, octyl formate, 2-pentylfuran, propanoic acid, butanoic acid, hexanoic acid, heptanoic acid, decanoic acid, 2-ethylhexanoic acid, carbon disulfide, 2-acetyl-2-thiazoline, benzothiazole, dimethyl sulfone, 3-methylthiopropanal, 2-butanone, acetoin, nerylacetone, 2,3-pentanedione, 2-nonanone, 3-undecanone, 6-methyl-5-hepten-2-one, 1-octen-3-one, 6-methyl-2-heptanone, γ-hexalactone, γ-octalactone, γ-nonanolactone, butyrolactone, 1,8-cineole, 2-phenoxyethanol, 2-methoxy-4-vinylphenol, (2E,4E)-nonadienal, (2E,4E)-decadienal, hexadecanal, 2-octenal, (2E,4E)-heptadienal, (2E)-nonenal, (2E)-heptenal, hexanal, nonanal, decanal, benzaldehyde, benzeneacetaldehyde, (2E)-undecenal, (2E)-decenal, dodecanal, acetaldehyde, furfural were purchased from Aladdin Biochemical Technology Co., Ltd. (Shanghai, China). Meanwhile, heptanal, octanal, 2-heptanone, 2-octanone, 3-octanone, dimethyl sulfide, pentanal, 2-methyl-3-heptanone, and n-alkanes (C7-C40) were supplied by Sigma-Aldrich (Shanghai, China). 2-Pentylfuran, 2-heptylfuran, 2-hexylfuran, and 2-butylfuran were of gas chromatography grade and obtained from Alfa Aesar (Shanghai, China). High-performance liquid chromatography (HPLC)-grade methanol was purchased from Merck (Darmstadt, Germany). Anhydrous ether, n-hexane, chloroform, and ammonium acetate were analytical-grade and purchased from Sinopharm Chemical Reagent Co., Ltd. (Beijing, China).

### 2.2. Sample Handling and SPME Procedure

Pork, beef, mutton, chicken, and duck breast were purchased from the local market. Those meats were minced, packaged in the plastic bag, and cooked in a water bath at 80 °C for 30 min. In addition, the cooked meat was cooled and ground in liquid nitrogen for the following SPME procedure.

SPME was directly performed in the TriPlus RSH autosampler (Thermo Fisher Scientific (Bremen, Germany)). The procedure was as follows: A 3 g minced sample was introduced in a 20 mL glass vial. The vials were immediately closed with a magnetic cap fitted with a polytetrafluoroethylene-silicone septum. The sample vial was incubated at 55 °C for 20 min and extracted at 55 °C for 40 min using a 50/30 μm Divinylbenzene/Carboxen/Polydimethylsiloxane (DVB/CAR/PDMS) fiber (Supelco, Inc., Bellefonte, PA, USA)). In order to ensure faster extraction, the vial was maintained in agitation during the extraction period. Once the extraction was finished, the fiber was automatically inserted into the injector and desorbed at 250 °C for 3 min. Between the consecutive analysis, the fiber was conditioned in the other injector port at 270 °C for 10 min.

### 2.3. Analysis of Volatile Compounds by GC-HRMS 

All analyses were conducted on a Q-Exactive Orbitrap mass analyzer equipped with a TriPlus RSH autosampler and Trace 1310 GC (Thermo Fisher Scientific, Bremen, Germany). A VF-WAX ms column (60 m × 0.25 mm i.d. × 0.25 μm film thickness, Agilent, Santa Clara, CA, USA) was used. Helium (99.9999%) with a constant flow rate of 1 mL/min was used as the carrier gas. The column oven was temperature-programmed starting at 40 °C for 2 min, then increased to 230 °C at a rate of 4 °C/min and then maintained at 230 °C for 5 min. Both of the transfer line 1 and transfer line 2 were set at 250 °C. MS was performed using electron impact ionization (EI) at 70 eV, operating in full scan mode at a resolving power of 60,000 full width at half maximum (FWHM). The scan range was from 30 to 400 m/z with an automatic-gain-control target value of 1E6. Ion source and transfer line temperatures for MS were set at 280 °C and 250 °C, respectively.

GC–MS data were acquired and processed using the Xcalibur 4.1 and TraceFinder 4.0 softwares (Thermo Scientific), respectively. Volatile compounds were identified in accordance with mass spectra and linear retention indices (LRIs) from NIST17 (v2.3) and the domestic library. The domestic library, namely the homeflavor library, was established using authentic reference standards, integrated with high resolution mass spectra and linear retention indices. Moreover, the high-resolution filtering (HRF) tool from the Tracefinder software was utilized to annotate every measured m/z peak and evaluate the mass accuracy of those ions when the NIST library was used. A series of standard alkanes (C7–C40; Sigma-Aldrich, St. Louis, MO, USA) were run under the same chromatographic conditions to calculate LRIs. 

### 2.4. Method Validation

Method validation was executed using 2-methyl-3-heptanone as the model compound. Linearity, sensitivity, accuracy, and precision were investigated. The calibration curve was plotted through the responses versus the concentrations. Sensitivity of the method was evaluated using the limit of detection (LOD, S/N = 3) and the limit of quantification (LOQ, S/N = 10) of 2-methyl-3-heptanone, calculated in light of the mutton with the lowest spiking level. 

### 2.5. Statistical Analysis

Alignment of mass signals (signal/noise≥ 3) was performed using Tracefinder software with the deconvolution plugin. Mass signals present in ≤4 replicates were discarded. For statistical analysis, the main tendencies of the generated data were compared and visualized using principal components analysis (PCA), volcano analysis, and partial least squares discriminant analysis (PLS-DA) after a log 10 transformation and Auto scaling of the samples using Metaboanalyst 5.0. Graphs were also produced using Microsoft Excel 16.30.

## 3. Results and Discussions

### 3.1. Optimization of Separation

Hydrocarbons, alcohols, aldehydes, ketones, furans, esters, sulfur-containing compounds, and other heterolytic compounds such as pyridines and pyrazines compose the main volatile compounds in different foods [23]. Complex compositions of volatiles would bring big challenges in their separation. Here, GC columns with stationary phases of nonpolar (5%-phenyl)-methylpolysiloxane and polar polyethylene glycol were compared. Apart from the boiling temperature, the polarity was another influencing factor in the separation mechanism for polar GC columns. This will lead to the decreased coelution and better separation of volatile compounds when the polar stationary phase of polyethylene glycol was used. In actuality, using mutton as the research model, the number of identified volatile compounds was increased by more than 50% when polar stationary phase polyethylene glycol was used, indicating that the polar stationary phase would be more suitable for volatiles’ profiling.

### 3.2. Optimization of Extraction

**Figure 1:**
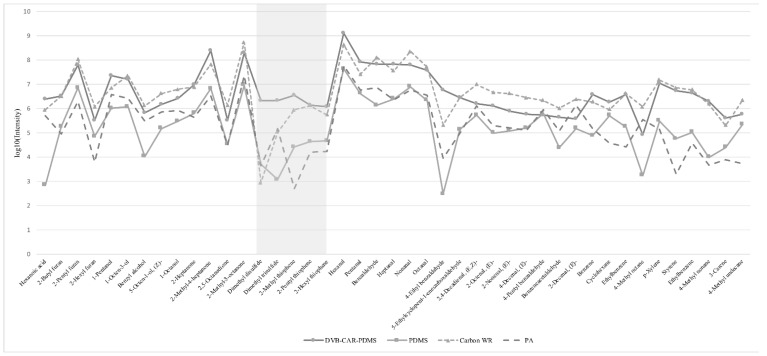
Intensities of typical volatiles detected using different SPME fibers. The shadow in means the emphasis on the sulphur-containing compounds.

SPME is one of the most widely used extraction methods for volatile compound analysis. Among the influencing parameters, the fiber type could be the most crucial one, which could significantly affect the profiling pattern [24]. The extracted compound types and their signal intensity would vary a lot according to the different fiber types. Totals of 85 μm Polyacrylate (PA), 50 μm/30 μm DVB/CAR/PDMS, 95 μm Carbon WR, and 30 μm PDMS were selected as the representative fibers for evaluating the extraction efficiency. Figure 1 shows the tendency of volatile compounds’ intensities with four kinds of fibers. Obviously, DVB/CAR/PDMS and Carbon WR showed better performance with higher signal intensities for most of the compounds. However, Carbon WR showed poor performance for sulfur-containing compounds. Considering that sulfur-containing compounds play pivotal roles in the meat flavor [25], DVB/CAR/PDMS would be selected for the further analysis. 

**Figure 2:**
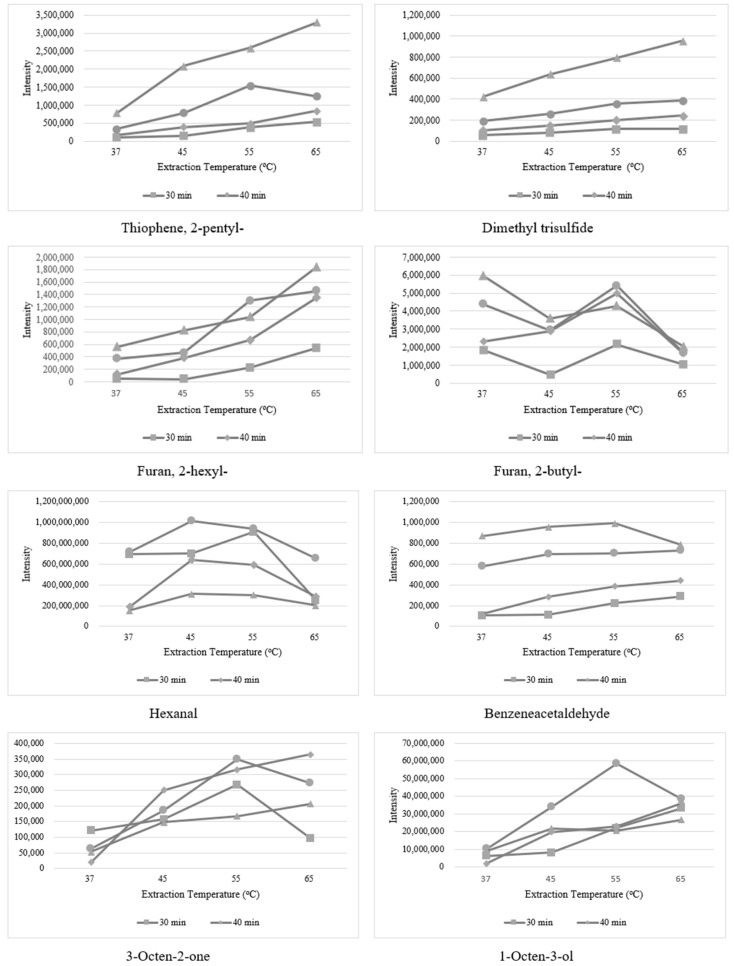
The influences of the extraction temperature and the extraction time on the intensities of some typical volatiles.

The equilibriums of volatile compounds among the fibers, the sample matrices, and the gas phase would determine the actions of volatile compounds in the extraction [13]. As for the extraction efficiency, the extraction temperature and the extraction time contributed a lot with a specified fiber [26]. Those two parameters were optimized comprehensively in this research. Figure 2 shows the influences of the extraction temperature and the extraction time on the intensities of the main volatile compounds in meat, including hydrocarbons, aldehydes, ketones, esters, furans, sulfur-containing compounds, and other heterolytic compounds. Basically, with the extraction time prolonged, the intensities of the typical volatile compounds were increased, especially for the aldehydes, furans, and sulfur-containing compounds. However, different situations would happen to some other compounds, such as ketones, alcohols, and hydrocarbons. Extraction for 60 min would give rise to the enhanced desorbing of those compounds. Taken together, 50 min would be chosen as the optimum extraction time in the profiling of volatile compounds. As for the extraction temperature, the intensities of the typical volatile compounds would reach the highest at 55 °C when the extraction lasted for 50 min, possibly because the desorbing would be the dominant action with the temperature continually increased. The duration of desorbing in the injector would be also highly associated with the sensitivity and the reproducibility. Here, the desorbing time ranging from 1 min to 7 min was investigated. 

**Figure 3:**
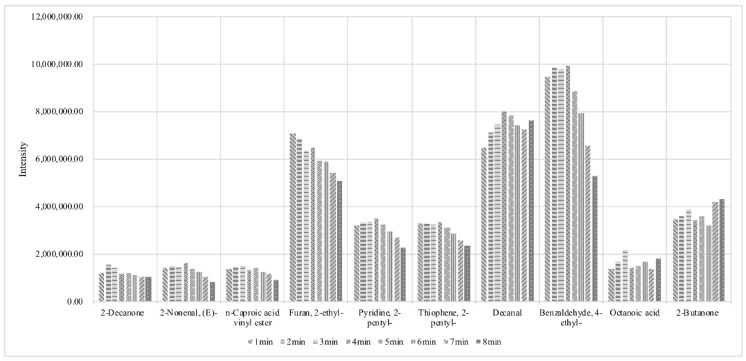
The influence of desorbing time on some typical volatiles.

As shown in Figure 3, with the increase in the desorbing time, the signal intensities for most of the compounds were steadily increased at the beginning. However, the intensities for some of the compounds were decreased when the desorbing was further proceeding. Therefore, the SPME conditions of incubation at 55 °C for 10 min, extraction at 55 °C for 50 min, desorbing at 270 °C for 3 min would be considered as the optimum.

### 3.3. Method Validation

Method validation was processed in the optimal conditions in order to investigate the applicability of the developed method. Linearity with the matrix-matched calibration curve for 2-methyl-3-heptanone was investigated and proved very well in the range of 0.33 to 33.33 μg/g with the linear regression correlation coefficient higher than 0.999, which is good for the quantitative analysis. LOD and LOQ were 0.01 and 0.03 μg/g, respectively. The precision was evaluated through spiking 2-methyl-3-heptanone (5 μL 50 μg/mL) in mutton. The RSD of six replications was less than 15%, indicating that the developed method was capable to be applied in the routine analysis. 

### 3.4. Identification of Volatile Compounds in Different Meats

The identification was processed through the MS pattern in NIST and homeflavor libraries, the retention index, and the HRF. When using the traditional NIST spectral matching at unit resolution, the HRF assigned tentative identification was based on the use of high-resolution mass spectra. The HRF scores obtained for the candidates should be higher than 95. The match factor based on the MS pattern should be higher than 750. Additionally, the difference in the retention index should be less than 20 for the homeflavor library while within 50 for the NIST library. It should be emphasized that the home-built library is necessary since the MS pattern could be changed according to the different MS analyzer. The fragments in the low-mass range would be lost for orbitrap MS. This would lead to the penalized score for the identification if the Nist library was used. With the optimum analysis conditions, five different kinds of boiled meats were analyzed, and 148 volatile compounds in total were identified as shown in Table 1 based on the above identification rules. Based on this simple cooking method at a low temperature for reflecting the original flavor of meats, the number of the detected volatiles was certainly large. Additionally, the final list of volatile compounds was used to run the statistical analysis for the assessment of the meat grouping and to determine differential volatile compounds.

It could be found that hydrocarbons, aldehydes, ketones, esters, acids, furans, sulfur-containing compounds, and lactones composed the main components of volatile compounds in meat from Table 1. Aromatic hydrocarbons including toluene, xylenes, and ethylbenzene were the dominant component, mainly coming from the feedstuffs [27]. The alkanals and alkenals such as hexanal, octanal, 2-decenal, and (2E,4E)-heptadienal were mainly formed through the autoxidation of the lipids [28]. The acetaldehyde and the aromatic aldehydes were produced through the Strecker degradation of amino acids and their derivatives, such as glycine, phenyl alanine, and so on [29]. Furans, alcohols, acids, and ketones were also from the autoxidation of the lipids with unsaturated fatty acids [30]. As for the sulfur-containing compounds, thiamine degradation, Maillard reaction, Strecker degradation, and its further oxidation could be their main formation routes [5,31,32]. Furaldehyde and their derivatives would be formed through the Maillard reaction [29]. Lactones are a group of cyclic esters and are formed by the intramolecular condensation of hydroxy fatty acids [33,34].

### 3.5. Statistical Analysis

**Figure 4:**
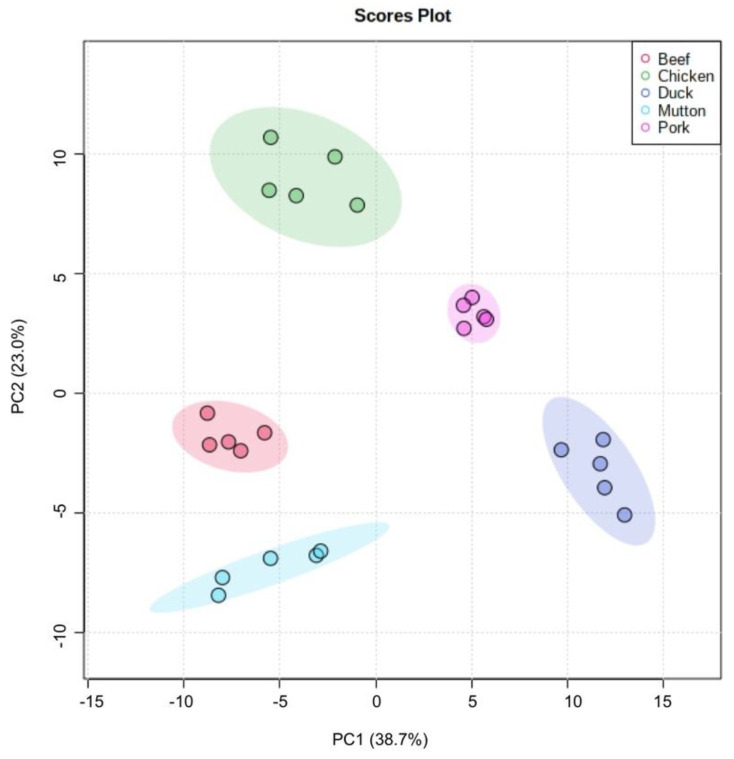
PCA plot according to the volatiles’ profiling in five different meats.

Volatilomics, utilizing the metabolomic pipelines, aims to characterize comprehensively a wide range of volatile molecules with the masses less than 550 Da, helps to compare the global profile of volatile compounds between groups of samples accurately, as well as identifies discriminatory compounds. PCA was performed to visualize how the volatile profiling differed between the five different meats (Figure 4). Based on all identified volatile compounds, the first two PCs, explaining over 60% of the total variance between the samples, showed a clear separation of the profiles of the five different meats. The clustering of the five replicate samples indicated that the applied analytical methods of GC-Q/orbitrap high resolution MS coupled with SPME were of good reproducibility and robustness, which are very important in untargeted studies for differentiation.

Another aim of this study was to define the specific compounds which are pivotal for the discrimination between the different meat samples. With the unpaired t-test, compounds responsible for the differences between the sample groups were found, as shown in Table 2. With the comparison of every two kinds of meats, dozens of compounds were present at abundances two times higher or lower (fold change > 2 or fold change < 0.5). In the comparison of mutton and beef meats, there were only 38 differentiating compounds found. In the formation pathways of meat aroma, lipid oxidation was one of the primary pathways. Saturated fatty acids (SFA) and monosaturated fatty acids (MUSFA) were the dominant fatty acids, and their levels in mutton and beef meats were similar [35]. In addition, the levels of polyunsaturated fatty acids (PUSFA) in both mutton and beef were relatively low. Therefore, this would lead to the similar pattern of volatile compounds produced through lipid autoxidation, resulting in the fewest differentiating compounds between mutton and beef. However, when comparing pork, duck, and chicken meats, the number of the differentiating volatile compounds would be elevated. It was convinced that PUSFAs [35], including linoleic acid, linolenic acid, and arachidonic acid, were the most easily autoxidized fatty acids. These fatty acids could produce complex patterns and large volumes of volatile compounds during heating procedures.

**Figure 5:**
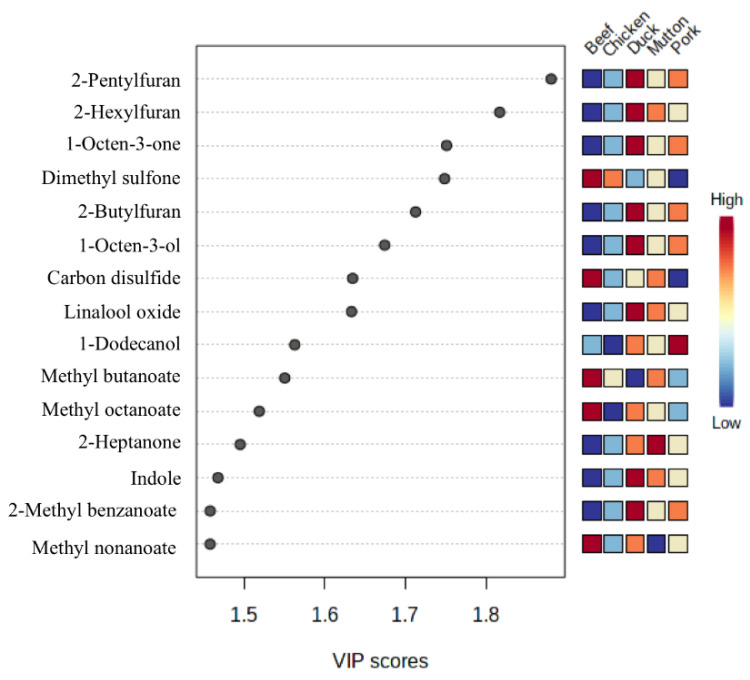
Variable importance in projection (VIP) score of volatile compounds.

From the results based on the PLS-DA analysis for the five meat categories, complete separation between every two meat categories was achieved. The model showed good interpretability, predictability, and non-overfitting due to the high value of R2 (cum, 98.6%) and Q2 (cum, 96.2%). Each meat category showed compact groups, indicating the authenticity of different meat species. In addition, 58 discriminant compounds were screened (VIP ≥ 1). Figure 5 shows 15 most important discriminant compounds. Interestingly, the levels of 2-pentyl furan, 2-butyl furan, 2-hexyl furan, 1-octen-3-ol, and 1-octen-3-one in duck meat, produced from the oxidation of n-6 fatty acids, were higher than those in the other four categories of meats. The highest levels of linoleic acid and arachidonic acid in duck meat [35] would contribute to the discrimination. In addition, carbon disulfide, dimethyl sulfoxide, fatty acid methyl esters including methyl butanoate, methyl octanoate, and methyl nonanoate were the most important discriminants for beef. Methyl esters would be produced through lipid degradation [36]. Carbon disulfide and dimethyl sulfoxide should come from the sulfur-containing amino acids such as methionine and cysteine.

Taken together, five categories of meat could be apparently distinguished according to the volatiles’ profiling. The profile pattern would vary a lot with specific volatiles generated from certain aroma-precursors such as lipids or amino acids. In addition, nowadays, volatile profiling combined with chemometric models show great potentials in protecting from food fraud, such as virgin olive oil [37] and honey [38]. Here, this study also gave a solid foundation to the meat differentiation as well as the meat authentication.

## 4. Conclusions

The present study developed an analytical strategy for the untargeted profiling of volatile compounds in boiled meat. The method based on the HS-SPME-GC-MS was investigated in detail, and the influencing parameters including extraction fibers, extraction temperatures, extraction time, and desorbing time were optimized comprehensively. High sensitivity and good reproducibility were achieved. For the confirmation of the volatiles, the combination of retention indices, the home-built high-resolution database (home flavor), NIST database matching using low-resolution mass spectrometry, and HRF scores based on the accurate masses was utilized. The home-built high-resolution database could significantly enhance the identification accuracy. The optimum untargeted approach in tandem with multivariate data analysis techniques was promising for meat categories’ discrimination and meat authentication. Duck, chicken, beef, mutton, and pork meats were easily distinguished based on their volatiles’ profiles. 2-Pentyl furan, 2-butyl furan, 2-hexyl furan, 1-octen-3-ol, and 1-octen-3-one were positively correlated with duck meat. Methyl esters would be the main discriminant biomarkers for beef. This phenomenon was caused by the different fatty acids’ pattern in different meat species.

## Figures and Tables

**Table 1:** Volatile compounds identified in five different meats.

Component Name	CAS Number	Retention Time (min)	Formula	RI	ΔRI	Identification Method
hydrocarbons						
(3Z)-Octene	14850-22-7	6.819	C_8_H_16_	858	0	RI ^a^, Nist, HRF ^b^
Decane	124-18-5	9.841	C_10_H_22_	1002	2	RI, Nist, HRF
(E,E)-1,3,5-Undecatriene	19883-29-5	22.704	C_11_H_18_	1390	2	RI, Nist, HRF
1,2,4-Trimethylbenzene	95-63-6	18.995	C_9_H_12_	1282	1	RI, Nist, HRF
1-Ethyl-3-methylbenzene	620-14-4	16.947	C_9_H_12_	1222	3	RI, Nist, HRF
1-Ethyl-2-methyl-benzene	611-14-3	18.294	C_9_H_12_	1261	3	RI, Nist, HRF
2-Methyl-5-(1-methylethyl)-bicyclo [3.1.0]hex-2-ene	2867-05-2	10.568	C_10_H_16_	1025	3	RI, Nist, HRF
1-Methyl-(1E)-propenyl- benzene	768-00-3	25.719	C_10_H_12_	1483	3	RI, Nist, HRF
(Z,E)-1,3,5-Undecatriene	19883-27-3	23.154	C_11_H_18_	1404	4	RI, Nist, HRF
Benzene	71-43-2	8.475	C_6_H_6_	944	5	RI, Homeflavor, HRF
2,2,4,6,6-Pentamethylheptane	13475-82-6	8.687	C_12_H_26_	954	5	RI, Nist, HRF
α-Methylstyrene	98-83-9	20.791	C_9_H_10_	1333	5	RI, Nist, HRF
Toluene	108-88-3	11.106	C_7_H_8_	1043	6	RI, Homeflavor, HRF
1,4-Diethylbenzene	105-05-5	19.642	C_10_H_14_	1299	6	RI, Nist, HRF
1-Ethyl-3,5-dimethylbenzene	934-74-7	20.517	C_10_H_14_	1325	6	RI, Nist, HRF
Ethylbenzene	100-41-4	13.572	C_8_H_10_	1122	7	RI, Nist, HRF
1-Ethyl-2,4-dimethylbenzene	874-41-9	21.536	C_10_H_14_	1355	7	RI, Nist, HRF
1,3-Dimethylbenzene	108-38-3	14.008	C_8_H_10_	1135	8	RI, Nist, HRF
p-Xylene	106-42-3	13.819	C_8_H_10_	1129	9	RI, Nist, HRF
Propylbenzene	103-65-1	16.311	C_9_H_12_	1203	9	RI, Nist, HRF
Mesitylene	108-67-8	17.607	C_9_H_12_	1241	10	RI, Nist, HRF
1-Methyl-4-propylbenzene	1074-55-1	19.843	C_10_H_14_	1306	10	RI, Nist, HRF
1,2,4,5-Tetramethylbenzene	95-93-2	24.442	C_10_H_14_	1443	10	RI, Nist, HRF
1-Methylethylbenzene	98-82-8	15.044	C_9_H_12_	1167	11	RI, Nist, HRF
4-Methylundecane	2980-69-0	15.393	C_12_H_26_	1178	31	RI, Nist, HRF
4-Octyne	1942-45-6	8.755	C_8_H_14_	956	44	RI, Nist, HRF
2-Ethyl-1,3-dimethylbenzene	2870-04-4	22.04	C_10_H_14_	1370	11	RI, Nist, HRF
Indane	496-11-7	22.21	C_9_H_10_	1376	11	RI, Nist, HRF
Styrene	100-42-5	18.196	C_8_H_8_	1258	12	RI, Homeflavor, HRF
p-Cymene	99-87-6	18.471	C_10_H_14_	1266	17	RI, Homeflavor, HRF
o-Xylene	95-47-6	15.512	C_8_H_10_	1181	18	RI, Homeflavor, HRF
dl-Limonene	138-86-3	15.761	C_10_H_16_	1187	24	RI, Homeflavor, HRF
alcohols						
1-Pentanol	71-41-0	18.029	C_5_H_12_O	1253	0	RI, Homeflavor, HRF
1-Octanol	111-87-5	27.993	C_8_H_18_O	1554	3	RI, Nist, HRF
(5Z)-Octen-1-ol	64275-73-6	29.75	C_8_H_16_O	1612	3	RI, Nist, HRF
1-Hexanol	111-27-3	21.337	C_6_H_14_O	1350	5	RI, Homeflavor, HRF
1-Octen-3-ol	53907-72-5	24.581	C_8_H_16_O	1446	5	RI, Homeflavor, HRF
1-Dodecanol	112-53-8	39.491	C_12_H_26_O	1964	5	RI, Homeflavor, HRF
α-Terpineol	98-55-5	32.337	C_10_H_18_O	1700	6	RI, Homeflavor, HRF
1-Undecanol	112-42-5	36.803	C_11_H_24_O	1861	9	RI, Nist, HRF
Benzenemethanol	100-51-6	37.313	C_7_H_8_O	1880	10	RI, Homeflavor, HRF
Benzeneethanol	60-12-8	38.257	C_8_H_10_O	1916	10	RI, Homeflavor, HRF
Phenol	108-95-2	40.655	C_6_H_6_O	2010	10	RI, Nist, HRF
5-Methyl-1-hexanol	627-98-5	24.695	C_7_H_16_O	1451	15	RI, Nist, HRF
3-Methylphenol	108-39-4	42.526	C_7_H_8_O	2086	13	RI, Homeflavor, HRF
esters						
Methyl hexadecanoate	112-39-0	45.681	C_17_H_34_O_2_	2222	0	RI, Homeflavor, HRF
Methyl nonanoate	1731-84-6	26.025	C_10_H_20_O_2_	1494	3	RI, Nist, HRF
Methyl propionate	554-12-1	7.671	C_4_H_8_O_2_	909	4	RI, Nist, HRF
Methyl 2-methylbutanoate	868-57-5	10.146	C_6_H_12_O_2_	1012	4	RI, Nist, HRF
Methyl decanoate	110-42-9	29.308	C_11_H_22_O_2_	1597	4	RI, Nist, HRF
Methyl octanoate	111-11-5	22.661	C_9_H_18_O_2_	1391	5	RI, Homeflavor, HRF
Ethyl acetate	141-78-6	7.291	C_4_H_8_O_2_	890	6	RI, Homeflavor, HRF
Methyl butanoate	623-42-7	9.485	C_5_H_10_O_2_	988	6	RI, Homeflavor, HRF
Methyl tetradecanoate	124-10-7	40.685	C_15_H_30_O_2_	2012	7	RI, Nist, HRF
Vinyl hexanoate	3050-69-9	16.871	C_8_H_14_O_2_	1220	13	RI, Homeflavor, HRF
Octyl formate	112-32-3	23.917	C_9_H_18_O_2_	1424	13	RI, Homeflavor, HRF
furans						
2-n-Butyl furan	4466-24-4	13.642	C_8_H_12_O	1124	1	RI, Nist, HRF
2-Hexylfuran	3777-70-6	20.606	C_10_H_16_O	1327	6	RI, Nist, HRF
2-n-Octylfuran	4179-38-8	27.509	C_12_H_20_O	1540	20	RI, Nist, HRF
2-n-Heptylfuran	3777-71-7	24.127	C_11_H_18_O	1433	12	RI, Nist, HRF
2-Pentylfuran	3777-69-3	17.014	C_9_H_14_O	1224	17	RI, Homeflavor, HRF
trans-2-(2-Pentenyl)furan	70424-14-5	19.597	C_9_H_12_O	1298	16	RI, Nist, HRF
acids						
Octanoic acid	124-07-2	41.818	C_8_H_16_O_2_	2059	1	RI, Nist, HRF
Pentanoic acid	109-52-4	33.403	C_5_H_10_O_2_	1739	6	RI, Nist, HRF
Decanoic acid	334-48-5	46.775	C_10_H_20_O_2_	2272	17	RI, Homeflavor, HRF
Hexanoic acid	142-62-1	36.336	C_6_H_12_O_2_	1846	18	RI, Homeflavor, HRF
Heptanoic acid	111-14-8	39.151	C_7_H_14_O_2_	1952	18	RI, Homeflavor, HRF
Propanoic acid	79-09-4	27.569	C_3_H_6_O_2_	1542	19	RI, Homeflavor, HRF
Butanoic acid	107-92-6	30.247	C_4_H_8_O_2_	1629	20	RI, Homeflavor, HRF
2-Ethylhexanoic acid	149-57-5	39.11	C_8_H_16_O_2_	1949	15	RI, Homeflavor, HRF
Benzoic acid	65-85-0	50.72	C_7_H_6_O_2_	2451	39	RI, Nist, HRF
sulfur-containing compounds						
Dimethyl sulfide	75-18-3	5.559	C_2_H_6_S	752	2	RI, Nist, HRF
Dimethyl sulfoxide	67-68-5	28.535	C_2_H_6_OS	1571	2	RI, Nist, HRF
Carbon disulfide	75-15-0	5.428	CS_2_	736	6	RI, Homeflavor, HRF
2-Acetyl-2-thiazoline	29926-41-8	34.315	C_5_H_7_NOS	1769	9	RI, Homeflavor, HRF
Benzothiazole	95-16-9	39.7	C_7_H_5_NS	1972	9	RI, Homeflavor, HRF
Dimethyl sulfone	67-71-0	38.08	C_2_H_6_O_2_S	1909	11	RI, Homeflavor, HRF
3-Methylthiopropanal	3268-49-3	25.067	C_4_H_8_OS	1462	7	RI, Homeflavor, HRF
ketones						
2-Pentanone	107-87-9	9.292	C_5_H_10_O	979	2	RI, Nist, HRF
3,5-Octadien-2-one	38284-27-4	27.051	C_8_H_12_O	1524	2	RI, Nist, HRF
2-Butanone	78-93-3	7.551	C_4_H_8_O	905	3	RI, Homeflavor, HRF
Acetoin	513-86-0	19.532	C_4_H_8_O_2_	1295	3	RI, Homeflavor, HRF
3-Nonanone	925-78-0	21.577	C_9_H_18_O	1357	3	RI, Nist, HRF
Nerylacetone	3879-26-3	36.726	C_13_H_22_O	1859	4	RI, Homeflavor, HRF
2,5-Octanedione	3214-41-3	20.45	C_8_H_14_O_2_	1324	5	RI, Nist, HRF
2,3-Pentanedione	600-14-6	11.567	C_5_H_8_O_2_	1059	6	RI, Homeflavor, HRF
2-Nonanone	821-55-6	22.72	C_9_H_18_O	1391	6	RI, Homeflavor, HRF
3-Undecanone	2216-87-7	28.345	C_11_H_22_O	1567	6	RI, Homeflavor, HRF
2-Heptanone	110-43-0	15.582	C_7_H_14_O	1183	7	RI, Homeflavor, HRF
6-Methyl-5-hepten-2-one	110-93-0	20.958	C_8_H_14_O	1339	7	RI, Homeflavor, HRF
1-Octen-3-one	4312-99-6	19.744	C_8_H_14_O	1302	8	RI, Homeflavor, HRF
6-Methyl-2-heptanone	928-68-7	17.46	C_8_H_16_O	1236	9	RI, Homeflavor, HRF
2-Octanone	111-13-7	19.141	C_8_H_16_O	1284	9	RI, Homeflavor, HRF
4-Octanone	589-63-9	17.097	C_8_H_16_O	1224	10	RI, Nist, HRF
3-Octanone	106-68-3	17.984	C_8_H_16_O	1253	11	RI, Homeflavor, HRF
aldehydes						
2-Methylbenzaldehyde	529-20-4	30.424	C_8_H_8_O	1636	4	RI, Nist, HRF
(2E,4E)-Nonadienal	5910-87-2	32.584	C_9_H_14_O	1709	4	RI, Homeflavor, HRF
(2E,4E)-Decadienal	25152-84-5	35.647	C_10_H_16_O	1819	4	RI, Homeflavor, HRF
Hexadecanal	629-80-1	43.834	C_16_H_32_O	2142	5	RI, Homeflavor, HRF
2-Octenal	2363-89-5	24.15	C_8_H_14_O	1434	6	RI, Homeflavor, HRF
(2E,4E)-Heptadienal	4313-03-5	26.297	C_7_H_10_O	1501	6	RI, Homeflavor, HRF
(2E)-Nonenal	18829-56-6	27.555	C_9_H_16_O	1541	6	RI, Homeflavor, HRF
(2E)-Heptenal	18829-55-5	20.629	C_7_H_12_O	1328	7	RI, Homeflavor, HRF
Nonanal	124-19-6	22.911	C_9_H_18_O	1395	7	RI, Homeflavor, HRF
Decanal	112-31-2	26.306	C_10_H_20_O	1501	7	RI, Homeflavor, HRF
Benzaldehyde	100-52-7	27.374	C_7_H_6_O	1534	7	RI, Homeflavor, HRF
Benzeneacetaldehyde	122-78-1	30.977	C_8_H_8_O	1654	7	RI, Homeflavor, HRF
(2E)-Undecenal	53448-07-0	34.04	C_11_H_20_O	1759	7	RI, Homeflavor, HRF
Pentanal	110-62-3	9.357	C_5_H_10_O	982	9	RI, Homeflavor, HRF
(2E)-Decenal	3913-81-3	30.846	C_10_H_18_O	1650	9	RI, Homeflavor, HRF
Dodecanal	112-54-9	32.741	C_12_H_24_O	1715	9	RI, Homeflavor, HRF
Heptanal	111-71-7	15.634	C_7_H_14_O	1182	10	RI, Homeflavor, HRF
Acetaldehyde	75-07-0	5.212	C_2_H_4_O	709	11	RI, Homeflavor, HRF
Octanal	124-13-0	19.251	C_8_H_16_O	1288	10	RI, Homeflavor, HRF
Hexanal	66-25-1	12.384	C_6_H_12_O	1084	14	RI, Homeflavor, HRF
2-Undecenal	2463-77-6	34.327	C_11_H_20_O	1771	20	RI, Nist, HRF
4-Pentylbenzaldehyde	6853-57-2	40.865	C_12_H_16_O	2019	16	RI, Nist, HRF
(2E,4Z)-Decadienal	25152-83-4	34.34	C_10_H_16_O	1771	17	RI, Nist, HRF
5-Ethylcyclopent-1-enecarboxaldehyde	36431-60-4	23.814	C_8_H_12_O	1424	14	RI, Nist, HRF
lactones						
γ-Hexalactone	695-06-7	32.725	C_6_H_10_O_2_	1715	8	RI, Homeflavor, HRF
γ-Octalactone	104-50-7	38.574	C_8_H_14_O_2_	1930	8	RI, Homeflavor, HRF
γ-Nonanolactone	104-61-0	41.418	C_9_H_16_O_2_	2043	8	RI, Homeflavor, HRF
Butyrolactone	96-48-0	30.624	C_4_H_6_O_2_	1642	10	RI, Homeflavor, HRF
others						
Furfural	98-01-1	25.323	C_5_H_4_O_2_	1470	7	RI, Homeflavor, HRF
5-Ethyl-2-furaldehyde	23074-10-4	30.713	C_7_H_8_O_2_	1645	0	RI, Nist, HRF
Butylated Hydroxytoluene	128-37-0	38.275	C_15_H_24_O	1917	8	RI, Nist, HRF
2,4-Di-tert-butylphenol	96-76-4	47.638	C_14_H_22_O	2309	9	RI, Nist, HRF
Camphor	76-22-2	27.144	C_10_H_16_O	1528	10	RI, Nist, HRF
1,8-Cineole	470-82-6	16.37	C_10_H_18_O	1206	11	RI, Homeflavor, HRF
2-phenoxyethanol	122-99-6	44.005	C_8_H_10_O_2_	2148	12	RI, Homeflavor, HRF
2-Methoxy-4-vinylphenol	7786-61-0	45.29	C_9_H_10_O_2_	2204	12	RI, Homeflavor, HRF
4-ethyl-1,2-dimethylbenzene	934-80-5	21.747	C_10_H_14_	1362	14	RI, Nist, HRF
Indole	120-72-9	50.997	C_8_H_7_N	2462	17	RI, Nist, HRF
2-hydroxybenzaldehyde	90-02-8	32.095	C_7_H_6_O_2_	1692	20	RI, Nist, HRF
2,6-Di-tert-butyl-4-hydroxy-4-methylcyclohexa-2,5-dien-1-one	10396-80-2	42.772	C_15_H_24_O_2_	2096	20	RI, Nist, HRF
trans-Linalool oxide	34995-77-2	25.392	C_10_H_18_O_2_	1473	21	RI, Nist, HRF
2,6,6-Trimethyl-1-cyclohexene-1-carboxaldehyde	432-25-7	30.337	C_10_H_16_O	1632	21	RI, Nist, HRF
(-)-Epicedrol	19903-73-2	43.728	C_15_H_26_O	2137	25	RI, Nist, HRF
Benzonitrile	100-47-0	29.92	C_7_H_5_N	1617	28	RI, Nist, HRF
2-Methylbenzonitrile	529-19-1	38.869	C_8_H_7_N	1940	28	RI, Nist, HRF
4-(5-Methyl-2-furanyl)-2-butanone	13679-56-6	32.083	C_9_H_12_O_2_	1691	14	RI, Nist, HRF
Formamide	75-12-7	34.621	CH_3_NO	1780	2	RI, Nist, HRF
4-Ethylpyridine	536-75-4	22.599	C_7_H_9_N	1386	1	RI, Nist, HRF
Isoquinoline	119-65-3	39.215	C_9_H_7_N	1953	4	RI, Nist, HRF
Tributyl phosphate	126-73-8	43.309	C_12_H_27_O_4_P	2121	7	RI, Nist, HRF
N,N-Dibutylformamide	761-65-9	34.483	C_9_H_19_NO	1776	19	RI, Nist, HRF
N-ethylbenzenamine	103-69-5	33.38	C_8_H_11_N	1736	13	RI, Nist, HRF

^a^ RI short for ”retention index”; ^b^ HRF short for “high resolution filtering”.

**Table 2:** Overview of the differentiating compounds, from volcano plot analysis with *p* < 0.05 and fold change ≥ 2.

Comparison Type	Number of Differentiating Compounds
beef vs. mutton	38
pork vs. mutton	58
pork vs. beef	57
beef vs. chicken	61
chicken vs. mutton	61
chicken vs. pork	46
duck vs. mutton	71
pork vs. duck	41
chicken vs. duck	73
beef vs. duck	74

## Data Availability

The datasets generated during and/or analyzed during the current study are available from the corresponding author on reasonable request.
